# Forecasting Stone-Free Status Following Percutaneous Nephrolithotomy Utilizing Explainable Machine Learning

**DOI:** 10.3390/jcm15041380

**Published:** 2026-02-10

**Authors:** Resul Çiçek, İbrahim Topçu, Bulut Dural, İpek Balıkçı Çiçek, Murat Yılmaz, Cemil Çolak

**Affiliations:** 1 Department of Urology, Faculty of Medicine, İnönü University, 1975 Malatya, Turkey; ibrahimtopcu15@hotmail.com (İ.T.); bulutdural@hotmail.com (B.D.); drmuratylmz023@gmail.com (M.Y.); 2 Department of Biostatistics and Medical, Faculty of Medicine, İnönü University, 1975 Malatya, Turkey; ipek.balikci@inonu.edu.tr (İ.B.Ç.); cemil.colak@inonu.edu.tr (C.Ç.)

**Keywords:** percutaneous nephrolithotomy, stone-free status, machine learning, XGBoost, explainable artificial intelligence

## Abstract

**Background:** This study aimed to create and evaluate explainable machine learning models for forecasting postoperative stone-free status following percutaneous nephrolithotomy (PNL) utilizing a substantial clinical cohort. **Methods:** This retrospective single-center analysis encompassed 2144 adult patients who received PNL from 2010 to 2024. We employed clinical, radiographic, stone-related, and surgical data to train four supervised machine learning models: Extreme Gradient Boosting (XGBoost), Random Forest, Light Gradient Boosting Machine (LightGBM), and Adaptive Boosting (AdaBoost). We used the Synthetic Minority Oversampling Technique exclusively on the training set to fix the class imbalance. We assessed the model’s accuracy, precision, recall, F1-score, and area under the receiver operating characteristic curve (ROC–AUC) to see how well it worked. SHapley Additive exPlanations (SHAP) were used to measure explainability. **Results**: The total stone-free rate was 84.8%. XGBoost had the best predictive performance of the models tested, with an accuracy of 0.916 and a ROC–AUC of 0.975. LightGBM was close behind. Random Forest and AdaBoost had relatively inferior performance. SHAP analysis identified anatomical anomalies as demonstrated the strongest association with stone-free outcomes. The size of the access sheath and the number of stones were next. Other parameters that were identified by SHAP as important contributors to model predictions were the placement of the stone, Guy’s Stone Score, the length of the operation, and the density of the stone. These feature associations demonstrated clinical coherence with established knowledge in surgical practice. **Conclusions**: Explainable machine learning algorithms, especially XGBoost, can accurately predict stone-free outcomes following PNL in a way that makes sense to doctors. The incorporation of SHAP improves transparency and facilitates the prospective application of these models as decision-support instruments in personalized surgical planning.

## 1. Introduction

All over the world, the incidence and prevalence of urolithiasis, a common urological condition, have been continuously rising. According to recent epidemiological studies, 9–10% of adults are afflicted, and a variety of geographic, demographic, and lifestyle factors affect the disease’s occurrence [1,2]. Notably, previous reports have suggested that the burden of urolithiasis in Turkey may be higher than in many other regions, potentially due to the combined effects of climatic conditions, dietary habits, and genetic predisposition [3].

Minimally invasive procedures play a crucial role in the surgical treatment of urolithiasis, and the size of the stone, its location, and the anatomy of the kidneys are the main factors influencing treatment plans. Percutaneous nephrolithotomy (PNL) is still one of the main and best surgical options for renal stones that are 2 cm or larger, according to the most recent guidelines from the European Association of Urology (EAU). Patients with staghorn calculi or a high total stone burden continue to benefit from PNL, especially when it comes to stone-free outcomes [4].

Following percutaneous nephrolithotomy (PNL), the stone-free rate (SFR) is considered the primary indicator of surgical success [5]. SFR is influenced by a range of clinical and radiological factors, including stone size, stone number, anatomical location, Guy’s Stone Score, Hounsfield unit (HU) values, the presence of anatomical anomalies, and the surgical technique employed [4].

The ability to predict stone-free status prior to percutaneous nephrolithotomy (PNL) is of critical importance for appropriate patient selection and the individualization of surgical strategy; accordingly, artificial intelligence- and machine learning-based models have attracted increasing attention in recent years [6]. Although conventional scoring systems such as Guy’s Stone Score, the S.T.O.N.E. score, and the CROES nomogram incorporate various anatomical and radiological parameters, their predictive accuracy for preoperative estimation of stone-free outcomes remains limited [7].

In a study published in 2022, a Random Forest model incorporating variables such as stone type, serum creatinine level, and hemoglobin was shown to predict stone-free outcomes with an accuracy of approximately 75% [8]. In another study from the same year, machine learning approaches, including LASSO-logistic regression, Naïve Bayes, and support vector machines, achieved a higher area under the curve (AUC) value of 0.879 compared to conventional scoring systems [9]. More recently, an analysis based on clinical and radiological data from 212 patients demonstrated that models such as XGBoost, Random Forest, and gradient boosting decision trees (GBDT) were able to predict stone-free outcomes with AUC values of up to 0.82 [10].

One of the main challenges limiting the clinical applicability of machine learning models is their lack of explainability. The use of opaque decision-making processes may undermine clinician confidence and restrict the integration of such models into routine clinical practice. To address this limitation, explainable artificial intelligence approaches, such as SHapley Additive exPlanations (SHAP), have been developed to provide detailed insights into how specific features contribute to model predictions, thereby enhancing transparency and interpretability [11]. Through these approaches, the influence of specific factors—such as stone density measured in Hounsfield units or a patient’s history of prior surgical interventions—can be directly visualized within the model’s decision process, thereby enhancing clinician confidence in machine learning-based predictions.

However, the current literature includes only a limited number of models that simultaneously achieve high predictive accuracy and provide meaningful interpretability for stone-free outcome prediction after PNL. Moreover, studies employing explainability techniques such as SHAP are predominantly based on small sample sizes and single-center datasets, which raises concerns regarding the generalizability of their findings.

The aim of this study was to evaluate the comparative performance of XGBoost, Random Forest, Light Gradient Boosting Machine (LightGBM), and Adaptive Boosting (AdaBoost) algorithms in predicting postoperative stone-free status after percutaneous nephrolithotomy (PNL) using multidimensional clinical and radiological data, and to enhance the interpretability of the best-performing model through SHAP analysis. Most existing models in the literature have primarily focused on a single algorithm, have addressed interpretability only to a limited extent, and have been developed using relatively small patient cohorts. In contrast, the present study not only provides a comprehensive comparative evaluation of multiple machine learning algorithms but also applies SHAP to transparently elucidate the decision-making process of the selected model. Consequently, the clinical and radiological parameters contributing most significantly to stone-free outcomes were identified both quantitatively and visually, thereby strengthening the biological and surgical interpretability of the proposed predictive framework.

Through this approach, the objective is not only to develop a predictive model with high accuracy but also to establish a clinically interpretable and reliable decision support system.

## 2. Materials and Methods

### 2.1. Study Design and Patient Population

This study was designed as a single-center, observational cohort study conducted at the Department of Urology, İnönü University Turgut Özal Medical Center. A total of 2144 adult patients who underwent percutaneous nephrolithotomy (PNL) between 2010 and 2024 were retrospectively identified through the institutional electronic medical record system. Patients younger than 18 years of age and those who underwent concomitant additional surgical procedures were excluded.

Sample size calculation was based on machine learning approach. A total of 2355 patients who underwent percutaneous nephrolithotomy were included in this study. Sample size adequacy was assessed using the events-per-variable (EPV) metric, which evaluates the ratio of outcome events to predictor variables in the model. Among 2144 patients, 326 (15.2%) experienced the minority outcome (residual stones/non-stone-free status). With 10 predictor variables included in the final models, the EPV was calculated as 32.6. This value substantially exceeds both the conventional minimum threshold of 10 EPV and the preferred benchmark of 20 EPV recommended for multivariable predictive modeling, indicating adequate sample size for stable parameter estimation and reduced overfitting risk in machine learning applications [12].

The study protocol was approved by the İnönü University Ethics Committee (approval number: 2025/8291), and all procedures were carried out in accordance with the principles of the Declaration of Helsinki.

### 2.2. Surgical Technique

All patients underwent percutaneous nephrolithotomy (PNL) under general anesthesia in the standard prone position. The procedure was initiated with cystoscopic placement of a 4–6 Fr ureteral catheter to facilitate retrograde opacification of the collecting system. Percutaneous access to the renal collecting system was achieved under fluoroscopic guidance through the appropriate calyx, selected according to stone location and renal anatomy. Following successful access, tract dilatation was performed in a stepwise manner based on patient- and stone-related characteristics, with the access sheath size determined at the discretion of the operating surgeon. Standard dilatation techniques were applied in all cases, and stone fragmentation was achieved using pneumatic and ultrasonic lithotripsy in accordance with established PNL techniques. At the end of the procedure, the renal collecting system was carefully inspected for residual fragments. The surgical approach and instrumentation were consistent with contemporary guideline-recommended practices for PNL [13,14].

At the end of the procedure, the renal calyces and pelvis were systematically inspected to identify any residual stone fragments. Postoperative stone-free status was defined as the absence of residual stones on non-contrast computed tomography, with fragments smaller than 4 mm considered clinically insignificant [15]. The decision to place a nephrostomy tube or to perform a tubeless PNL was made based on the patient’s intraoperative findings, estimated blood loss, and overall clinical condition. All patients were followed postoperatively according to a standardized institutional protocol, and complete clinical and radiological follow-up data were obtained in accordance with established guideline-based recommendations [13].

### 2.3. Variables and Definitions

The primary output variable of the study was postoperative stone-free status, which was defined as a binary outcome (presence vs. absence of residual stones) based on postoperative radiological assessment. Stone-free status was defined as the absence of residual stones on non-contrast computed tomography (CT), excluding fragments smaller than 4 mm that were considered clinically insignificant. Residual stone assessment was performed by inspection of the renal calyces and pelvis at the end of the procedure and confirmed by postoperative imaging.

The selection of predictor variables was guided by their established clinical relevance and their guideline-supported association with stone-free outcomes and perioperative complexity in percutaneous nephrolithotomy. Specifically, variable selection was aligned with the European Association of Urology (EAU) Guidelines on Urolithiasis, which highlight patient-related factors, stone burden characteristics, anatomical considerations, and procedural parameters as the principal determinants of surgical success and complication risk following PNL [16]. 

In addition to guideline-based justification, the inclusion of these variables was further supported by evidence from recent large-scale clinical studies and machine learning-based analyses, which have consistently demonstrated their predictive value for postoperative stone-free status and operative outcomes. Collectively, this evidence provides a robust rationale for the integration of these variables into multivariable predictive modeling frameworks [14].

Predictor variables were evaluated across three main domains:

(a) Patient-related variables: age, sex, American Society of Anesthesiologists (ASA) physical status score, and the presence of comorbid conditions, including hypertension, diabetes mellitus, and coronary artery disease.
(b) Stone-related variables: stone morphology (single stone, multiple stones, partial staghorn, and complete staghorn calculi), stone number, stone location, stone density measured in Hounsfield units (HU), true stone surface area (mm^2^), and Guy’s Stone Score. Stone surface area was defined as the two-dimensional measurement calculated from axial CT images, whereas true stone surface area represented the cumulative surface area calculated across all CT slices to more accurately reflect total stone burden.
(c) Surgery-related variables: access sheath size (Fr), calyx of entry, operative time (minutes), application of a tubeless PNL approach (yes/no), and the need for additional intraoperative interventions.

Anatomical anomalies were defined as congenital renal or collecting system abnormalities that could increase the technical complexity of PNL, including horseshoe kidney, renal malrotation, duplex collecting system, and ureteropelvic junction obstruction.

All preoperative stone characteristics were assessed using non-contrast CT. Stone surface area and HU measurements were calculated using automated measurement tools in accordance with standardized radiological protocols. All imaging assessments were performed independently by two experienced uro-radiologists.

Postoperative stone-free status was assessed using non-contrast computed tomography performed between postoperative days 1–3 (median: day 2) before hospital discharge. This early imaging protocol was consistently applied throughout the study period to ensure standardized outcome assessment and minimize loss to follow-up.

### 2.4. Modelling

#### 2.4.1. Data Preprocessing

The dataset was prepared for predictive modeling using a standardized preprocessing pipeline. Categorical variables were converted into numerical representations through label encoding. To maintain cohort size and ensure model stability, missing values in continuous variables were imputed using the mean value of the corresponding feature. Importantly, all preprocessing procedures, including encoding and imputation, were performed after the train–test split to prevent information leakage and ensure unbiased model evaluation [17].

#### 2.4.2. Class Imbalance Management

The original dataset exhibited substantial class imbalance with 354 patients (15.0%) classified as Class 0 (residual stone/incomplete clearance) and 2001 patients (85.0%) classified as Class 1 (complete stone-free clearance). To address this imbalance, Synthetic Minority Oversampling Technique (SMOTE) was applied to the entire dataset prior to train-test splitting. Following SMOTE application, the dataset was balanced to 2001 Class 0 cases and 2001 Class 1 cases (50:50 distribution), resulting in a total dataset size of 4002 samples.

#### 2.4.3. Feature Selection Strategy

To improve interpretability and reduce dimensionality, feature selection was performed on the training dataset using a Random Forest-based feature-importance ranking. The most informative predictors were retained for subsequent model development and were used consistently across all algorithms. This approach effectively balances model complexity with clinical interpretability while identifying variables that demonstrate the strongest associations with stone-free outcomes. The selected features encompassed clinically relevant variables across patient demographics, stone characteristics, and operative parameters, ensuring that essential prognostic information was preserved for accurate outcome prediction.

#### 2.4.4. Model Evaluation and Selection: Machine Learning Model Development

Four supervised machine learning algorithms—Extreme Gradient Boosting (XGBoost), Random Forest, Light Gradient Boosting Machine (LightGBM), and Adaptive Boosting (AdaBoost)—were trained to predict postoperative stone-free status. The dataset was split into training (80%) and testing (20%) subsets using stratified sampling to maintain outcome proportions. Model training was conducted with five-fold cross-validation within the training set to obtain robust performance estimates and limit overfitting [18].

Hyperparameters were optimized empirically through randomized search with 5-fold cross-validation conducted exclusively within the training dataset to prevent information leakage to the test set. Optimization ranges included: XGBoost (learning_rate: 0.01–0.2, max_depth: 3–9, n_estimators: 50–200, subsample: 0.7–1.0, colsample_bytree: 0.7–1.0); Random Forest (n_estimators: 50–200, max_depth: 5–15, min_samples_split: 2–10, min_samples_leaf: 1–4); LightGBM (learning_rate: 0.01–0.2, max_depth: 3–9, n_estimators: 50–200, subsample: 0.7–1.0, feature_fraction: 0.7–1.0); and AdaBoost (n_estimators: 50–150, learning_rate: 0.8–1.2). Optimal hyperparameters were selected based on mean ROC–AUC across cross-validation folds.

The models were configured with the following hyperparameters:

- XGBoost was configured with 100 estimators, maximum tree depth of 6, learning rate of 0.1, subsample ratio of 0.8, and feature subsample ratio (colsample_bytree) of 0.8, using binary logistic objective function with logarithmic loss evaluation metric.
- Random Forest consisted of 100 decision trees with maximum depth of 10, minimum samples required to split an internal node of 5, and minimum samples per leaf of 2.
- LightGBM utilized 100 estimators with maximum tree depth of 6, learning rate of 0.1, subsample ratio of 0.8, and feature fraction of 0.8, employing binary objective with binary logarithmic loss metric.
- AdaBoost was configured with 100 estimators and learning rate of 1.0.

All models were initialized with random state 42 to ensure reproducibility of results. Model performance on the test set was assessed using accuracy, precision, recall, F1-score, and the area under the receiver operating characteristic curve (ROC-AUC), with 95% confidence intervals calculated for all performance metrics. The final model was selected primarily based on accuracy while also considering the overall balance and consistency of performance metrics.

#### 2.4.5. Explainable Artificial Intelligence Analysis

To enhance clinical interpretability, SHapley Additive exPlanations (SHAP) were applied to the best-performing model. SHAP values were used to quantify both global feature importance and individual-level feature contributions, thereby enabling transparent interpretation of how specific clinical, radiological, and operative variables influenced the predicted probability of postoperative stone-free status. This approach facilitates insight into model behavior at both the population and patient levels, supporting clinically meaningful interpretation of the model’s predictions [19].

## 3. Results

### 3.1. Baseline Patient Characteristics and Stone-Free Outcomes

A total of 2144 adult patients who underwent percutaneous nephrolithotomy (PNL) were included in the final analysis. The mean age was 49.3 ± 15.2 years (range: 18–90), and 61.0% of the cohort was male. According to the American Society of Anesthesiologists (ASA) classification, 54.5% of patients were ASA III, while 28.7% and 16.8% were ASA I and ASA II, respectively.

The overall complete stone-free rate was 84.8%. Clinically insignificant residual fragments were detected in 8.0% of patients, whereas clinically significant residual stones were observed in 7.1% (Table 1).

### 3.2. Stone Burden and Surgical Features

The mean stone count was 2.16 ± 1.48, with a mean stone surface area of 637.2 ± 432.8 mm^2^ and a mean stone density of 836.2 ± 151.0 Hounsfield units. Guy’s Stone Score distribution revealed that 35.5% of patients were classified as GSS I, 45.7% as GSS II, 7.4% as GSS III, and 11.4% as GSS IV. Partial or complete staghorn calculi were present in 18.8% of cases.

From a surgical perspective, the mean access sheath size was 24.4 ± 2.7 Fr, and the mean operative time was 60.4 ± 23.9 min. Lower calyx access was the most common entry site (70.4%). A second access tract was required in 4.8% of cases. Anatomical anomalies were identified in 18.5% of patients. The mean length of hospital stay was 1.99 ± 1.06 days (Table 2).

### 3.3. Feature Selection and Model Performance

Prior to model development, feature selection was performed using a Random Forest-based importance ranking. Ten variables with the highest predictive contribution to stone-free status were retained, including stone localization, stone count, stone surface area, Hounsfield unit value, Guy’s Stone Score, anatomical anomaly, sheath size, stent usage, operative duration, and true stone surface area.

**Figure 1:**
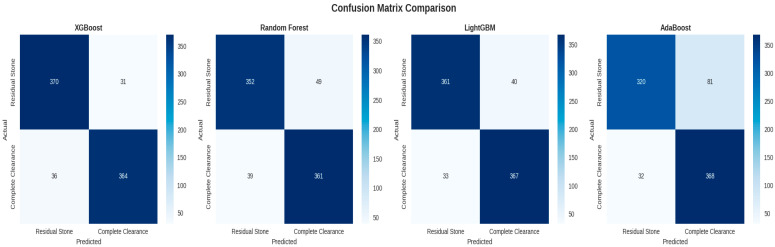
The Confusion Matrices of the Models.

Using the selected features, four machine learning algorithms (XGBoost, Random Forest, LightGBM, and AdaBoost) were trained and evaluated. The confusion matrices of the models are presented in Figure 1, and comparative performance metrics are summarized in Table 3.

### 3.4. Comparative Model Evaluation

A comparative analysis of the evaluated machine learning algorithms demonstrated that XGBoost attained the highest overall predictive performance, achieving an accuracy of 0.9164 and a ROC-AUC of 0.9746, which signifies exceptional discriminative capability for predicting postoperative stone clearance outcomes. LightGBM exhibited comparable robust performance (accuracy: 0.9089, ROC-AUC: 0.9719) and was further distinguished by the lowest cross-validation standard deviation among the examined models, thereby indicating superior robustness and stability across varying data partitions.

Conversely, the Random Forest and AdaBoost algorithms yielded comparatively inferior performance metrics, reflecting diminished predictive accuracy and reduced discriminative capacity relative to the gradient boosting-based methodologies under consideration.

### 3.5. Receiver Operating Characteristic (ROC) Curve Analysis

**Figure 2:**
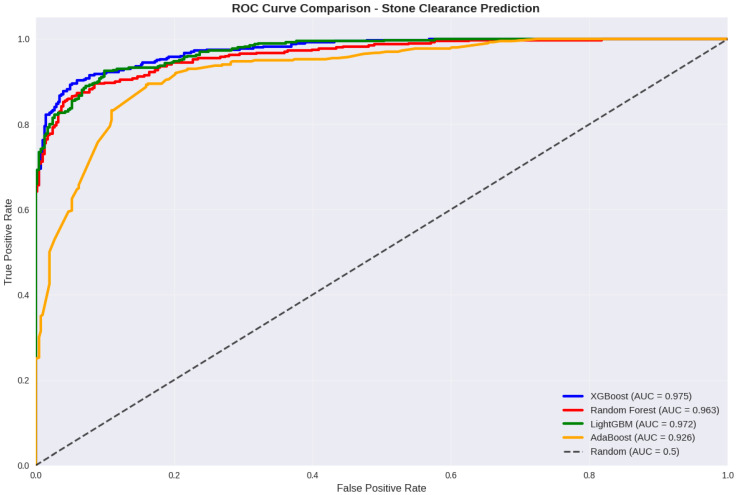
ROC curves of the evaluated machine learning models.

The discriminative performance of the developed machine learning models was further evaluated using ROC curve analysis. As illustrated in Figure 2, all models demonstrated strong classification capability, with ROC curves positioned well above the diagonal reference line representing random classification.

Among the evaluated algorithms, the XGBoost model achieved the highest area under the ROC curve (AUC = 0.975), indicating excellent ability to distinguish between patients with and without postoperative stone clearance. Light Gradient Boosting Machine (LightGBM) followed closely with an AUC value of 0.972, while the Random Forest model also showed robust discriminative performance (AUC = 0.963). In contrast, the AdaBoost model exhibited comparatively lower, though still acceptable, predictive performance (AUC = 0.926, Figure 2).

Overall, ROC curve analysis confirmed that all evaluated models possessed high sensitivity–specificity trade-offs across a wide range of decision thresholds. The superior AUC observed for the XGBoost model supports its selection as the primary algorithm for subsequent explainable artificial intelligence analysis.

### 3.6. Model Calibration and Clinical Utility Assessment

**Figure 3:**
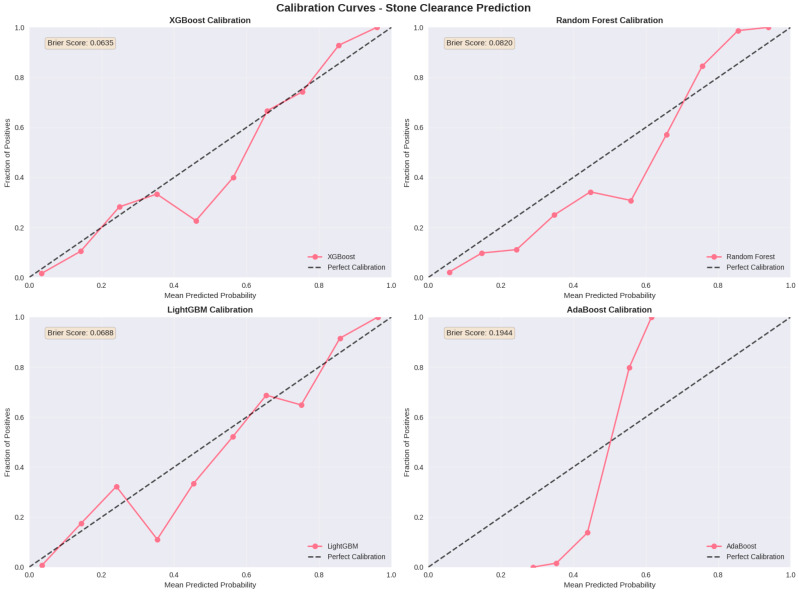
Calibration curves for stone-free status prediction models.

In addition to discrimination metrics, model calibration was comprehensively assessed to determine whether predicted probabilities aligned with observed outcome frequencies. Calibration curves were constructed through partitioning of the test dataset into deciles of predicted probability risk, with mean predicted probabilities compared against observed outcome proportions within each decile. The XGBoost model demonstrated good calibration, with a Brier score of 0.0635, indicating minimal systematic discrepancy between predicted and actual probabilities. This metric is particularly clinically relevant, as it quantifies the mean squared error of probability predictions; a low Brier score indicates that model-generated predicted probabilities can be reliably interpreted and communicated to patients during preoperative risk stratification. LightGBM exhibited comparable calibration performance (Brier score: 0.0688), whereas Random Forest (Brier score: 0.0820) and AdaBoost (Brier score: 0.1944) demonstrated progressively greater calibration error. The superior calibration characteristics of gradient boosting-based algorithms further substantiated the selection of XGBoost as the primary clinical decision-support framework (Figure 3).

**Figure 4:**
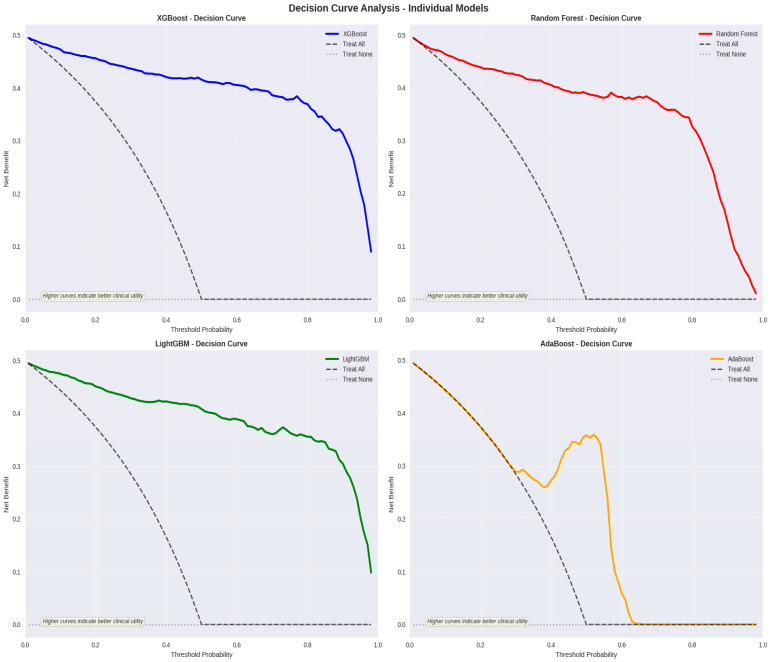
Decision curve analysis (DCA) for stone-free status prediction models.

To evaluate whether predictions derived from the XGBoost model would provide clinical utility for surgical decision-making, decision curve analysis (DCA) was performed across a range of threshold probabilities (0–100%). DCA quantifies net benefit—mathematically defined as the difference between the weighted true positive rate and weighted false positive rate, with weighting determined by the clinical threshold probability at which intervention is recommended. Across the clinically relevant threshold probability range (0–60%), the XGBoost model generated substantially greater net benefit compared with both “treat all” (assume all patients will achieve stone-free outcomes) and “treat none” (assume no patient will achieve stone-free outcomes) strategies, indicating that clinical decisions informed by model predictions would yield superior outcomes compared with default treatment assumptions. Specifically, at a threshold probability of 20% (representing a conservative clinical threshold), the XGBoost model provided incremental net benefit of approximately 15–20% compared with the “treat all” strategy. This clinical benefit was maintained across higher threshold probabilities (40–60%), supporting the model’s utility for diverse risk-stratification scenarios. The decision curves for XGBoost demonstrated superior performance compared with alternative algorithms, further justifying its selection for clinical implementation (Figure 4).

Collectively, these calibration and clinical utility analyses established that the XGBoost model possessed not only excellent discriminative capability (ROC–AUC: 0.975) but also demonstrated clinically actionable predictive accuracy with preserved probability calibration across the test cohort. The convergence of high discrimination, favorable calibration, and positive net benefit across clinically relevant decision thresholds indicates that the model demonstrates sufficient performance characteristics for potential application as a clinical decision-support system.

### 3.7. Explainable Artificial Intelligence (SHAP) Analysis

To enhance the interpretability of the predictive framework, SHAP analysis was applied to the best-performing XGBoost model. Global feature importance, quantified using mean absolute SHAP values, identified anatomical anomaly as the most influential predictor of postoperative stone clearance. This variable demonstrated a markedly higher contribution compared with all other features, underscoring the dominant impact of renal and collecting system anatomy on surgical outcomes.

Following anatomical anomaly, access sheath size and stone count emerged as the second and third most impactful variables, respectively. These findings indicate that both surgical access characteristics and overall stone burden play a critical role in shaping model predictions. Stone localization and Guy’s Stone Score contributed at a moderate level, supporting the clinical relevance of stone complexity and intrarenal distribution even within an advanced machine learning framework.

Operative duration, stone density expressed as Hounsfield Unit (HU) value, and stone surface area exhibited comparable, intermediate SHAP contributions, suggesting that procedural complexity and radiological stone properties exert a cumulative but less dominant influence on stone clearance probability. In contrast, stent usage and true stone surface area showed the lowest SHAP values, indicating a relatively limited independent contribution to the model’s decision-making process.

**Figure 5:**
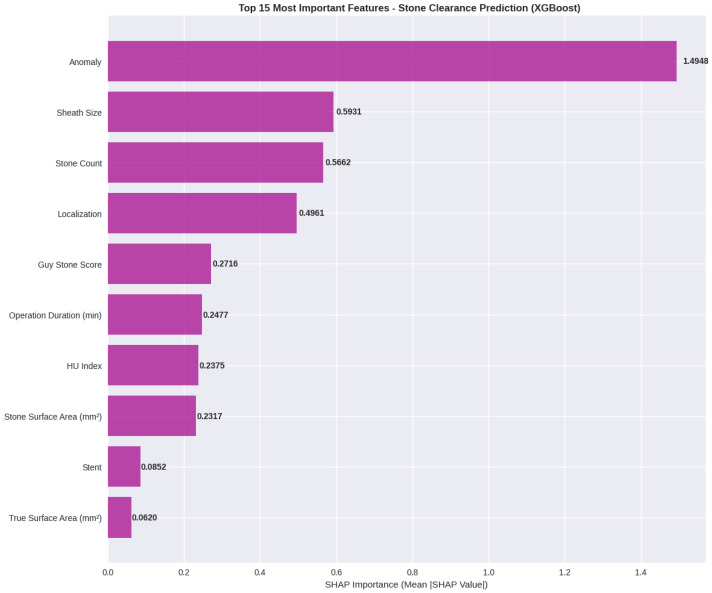
SHAP Summary Plot for XGBoost Model: Feature Importance Analysis in Stone Clearance Prediction.

**Figure 6:**
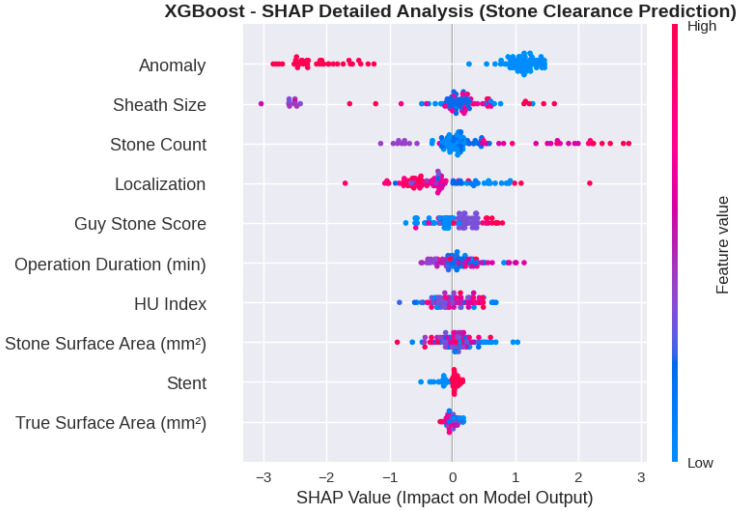
SHAP summary plot illustrating the direction and magnitude of feature effects for the XGBoost model.

The SHAP summary and detailed plots (Figure 5 and Figure 6) further illustrated both the magnitude and direction of feature effects at the individual patient level. High and low feature values were associated with positive or negative SHAP values depending on the variable, reflecting non-linear and patient-specific interactions within the model. This approach enables transparent interpretation of how clinical, radiological, and surgical factors collectively influence model outputs, thereby strengthening the clinical interpretability and credibility of the XGBoost-based prediction model.

## 4. Discussion

The findings of the present study align with and extend existing literature demonstrating the utility of machine learning models for predicting stone-free outcomes after PNL. Previous investigations have reported moderate to good predictive performance using algorithms such as XGBoost, Random Forest, and support vector machines, with AUC values generally ranging between 0.82 and 0.85 in relatively small cohorts and, in some cases, external validation settings [19,20]. More recently, the integration of radiomic features with clinical variables has been shown to further enhance model performance, highlighting the added value of advanced imaging-derived data beyond conventional stone characteristics [10]. Additionally, comparative analyses of multiple boosting-based algorithms have suggested that model selection and optimization strategies substantially influence discriminative ability, with reported AUC values approaching 0.90 in selected settings [21]. In contrast to prior studies, the present analysis leverages a substantially larger clinical cohort and incorporates explainable artificial intelligence techniques, thereby providing both high predictive performance and transparent interpretation of model behavior, which may facilitate broader clinical applicability. Notably, our cohort demonstrated a mean stone density of 836.2 ± 151.0 HU, with no stones exceeding 1000 HU. This distribution suggests a predominance of calcium oxalate and mixed-composition stones, with relatively few cystine or brushite calculi. Stone composition analysis was not routinely performed in our cohort, which represents a limitation. Future models incorporating biochemical stone analysis may provide enhanced predictive accuracy, particularly for harder stone types.

The data from our study strongly demonstrate the potential of ML models in predicting stone-free status after PNL and show findings comparable to those reported in similar studies in the literature. In a study published in 2023, a cohort of 320 patients was used to develop XGBoost, RF, and SVM models; the XGBoost model achieved an AUC of 0.84 on external validation. This study demonstrated that machine learning models can outperform conventional scoring systems such as the Guy’s Stone Score and the S.T.O.N.E. score in predicting stone-free outcomes [20]. Additionally, in a model developed by Fan et al. to predict residual stone and recurrence rates in patients undergoing PNL in the lateral decubitus position, the XGBoost algorithm achieved an accuracy of 86.8% in predicting stone residuals [21]. This also suggests that our model has the potential to achieve a high level of accuracy comparable to those reported in the existing literature.

In another study conducted by Zou et al., a model developed by integrating clinical variables with radiomic imaging features achieved an AUC of 0.85 for predicting stone-free status [10]. This approach highlights the added value of imaging-derived information to predictive models and illustrates how radiomic parameters, which were not included in our analysis, may further enhance model performance. In a study published in 2025, Liu et al. reported that among models developed using Gradient Boosting Decision Trees, XGBoost, and Random Forest algorithms, the GBDT model demonstrated particularly strong performance, achieving high accuracy and near-optimal discriminative ability with AUC values approaching 0.90 [22]. Our findings indicate that the choice of model and the optimization strategies applied have a substantial impact on predictive performance.

The SHAP-based analysis represents one of the most important contributions of this study. SHAP enables quantitative assessment of each variable’s contribution to the model’s predictions at both the global and individual levels, thereby providing transparent and interpretable insight into the decision-making process of the model [23]. Lundberg and Lee defined the SHAP framework as a unifying approach within the class of additive feature importance methods and showed that, owing to its consistency properties, it is ideal for explaining predictive models [11]. The perception of machine learning models as “black boxes” poses a significant barrier to their adoption in clinical practice. The use of SHAP allows clinicians to understand the rationale behind specific model predictions, enabling more confident interpretation and application of the results. For example, Feretzakis et al. demonstrated that enhancing model interpretability through SHAP can provide meaningful support for clinical decision-making systems [23]. This method facilitates the identification and understanding of important variables within highly complex feature sets commonly encountered in healthcare data.

From a variable-level clinical perspective, the feature-importance pattern identified by SHAP analysis closely aligns with well-established determinants of stone-free outcomes following percutaneous nephrolithotomy. Anatomical anomalies emerged as the most influential negative predictor, a finding that is consistent with evidence from large endourological series reporting lower stone-free rates and increased procedural complexity in patients with horseshoe kidneys, renal malrotation, and duplex collecting systems, even when procedures are performed in high-volume centers [24,25]. Access sheath size emerged as the second most influential variable in the SHAP analysis, reflecting the well-recognized balance between improved visualization and more efficient fragment evacuation associated with larger tract diameters, and the concomitant increase in bleeding risk. Previous comparative studies have reported higher stone clearance rates with larger access tracts, particularly in patients with a high stone burden, supporting the clinical relevance of this finding [26,27]. Stone number and overall stone burden were associated with a reduced probability of achieving stone-free status, consistent with evidence from large multicenter analyses demonstrating that stone multiplicity and staghorn morphology remain among the strongest predictors of residual fragments following percutaneous nephrolithotomy [28,29]. Moderate contributions from stone location and Guy’s Stone Score further underscore the importance of intrarenal stone distribution and anatomical complexity, consistent with findings from external validation cohorts showing progressive declines in stone-free rates with increasing Guy’s Stone Score grades [30]. Operative duration and stone density measured in Hounsfield units demonstrated consistent but secondary associations with stone-free outcomes, suggesting that procedural efficiency and stone composition may influence surgical success indirectly through fragmentation difficulty rather than serving as primary limiting factors [31,32]. Collectively, the concordance between SHAP-derived feature importance and high-quality clinical evidence reinforces the biological plausibility of the model and supports its interpretability as a clinically meaningful decision-support framework rather than a purely data-driven black box.

In our study, the identification of anatomical anomalies, sheath size, and stone number as highly important variables by the model is also clinically consistent. The literature frequently emphasizes that anatomical variations can increase surgical complexity and negatively affect stone clearance outcomes [24]. In addition, the impact of operative parameters—such as sheath size, operative time, and energy utilization—has also been shown to play a significant role in similar studies. For example, in a predictive modeling study, stone burden and stone-free status emerged as prominent variables in estimating PNL operative duration [33]. This finding supports that the model’s sensitivity to operative factors is well aligned with real-world clinical practice. It should be noted that several studies referenced in our comparative analysis, including conventional scoring systems and some machine learning models, included patients treated in supine or lateral positions. Positional differences may influence surgical access, stone clearance efficiency, and complication profiles. Our model, developed exclusively from prone-position PNL data, may require position-specific recalibration when applied to supine or lateral decubitus cohorts [34,35]. Therefore, the choice of patient position in the surgical approach should be evaluated together with factors such as the location of the stone, the patient’s condition, and the surgeon’s experience.

### Limitations and Future Directions

Nevertheless, the model has certain limitations with respect to generalizability and clinical applicability. First, the reliance on a single-center dataset suggests that model performance may vary across different patient populations. Geraghty et al. have reported that machine learning models validated over time provide more reliable results compared with those developed using single-center data alone [6]. In this context, testing the model on independent datasets obtained from different centers through external validation is necessary. Second, the absence of additional imaging-derived variables—such as radiomic features, three-dimensional stone morphometry, tissue heterogeneity, and stone surface texture—may have limited the model’s predictive power. The study by Zou et al. demonstrated that integrating radiomic features into predictive models can lead to improvements in AUC performance [10]. Third, even when imbalance-handling techniques such as SMOTE are applied, the risk of overfitting must always be carefully considered. Meticulous tuning of model hyperparameters and rigorous implementation of cross-validation strategies are essential to mitigate this risk. In addition, evaluating model performance in terms of classification errors—particularly false-positive and false-negative rates—and examining the potential impact of these errors on surgical outcomes are of critical importance. In addition, it should be acknowledged that surgical techniques, device technologies, and operator experience may evolve over time. The equipment and protocols used during the period in which the model was trained may differ from those employed in future surgical practice; therefore, strategies for periodic retraining and model adaptation should be considered. Moreover, to enable practical clinical integration of machine learning models, cost–benefit analyses are warranted, and factors such as ease of use, computational resource requirements, and compatibility with existing clinical workflows should be carefully evaluated. In addition, Sensitivity analyses comparing multiple imputation strategies (e.g., MICE, KNN imputation) may be conducted in future validation studies to assess the robustness of our findings across different missing data handling approaches. All procedures were performed in the standard prone position without combined endoscopic approaches. The absence of combined endoscopy (such as simultaneous retrograde intrarenal surgery) may have influenced stone-free rates, as hybrid techniques have been shown to improve clearance in select complex cases. Future studies incorporating combined approaches would provide valuable comparative data. The study period spanned 14 years (2010–2024), during which lithotripsy technology and disposable instruments evolved considerably. Advancements in laser technology, ultrasonic lithotripters, and access sheath design may have influenced stone fragmentation efficiency and clearance rates over time. This temporal variation represents a potential confounding factor that should be addressed through periodic model retraining and validation across different technological eras.

## 5. Conclusions

In this study, multiple machine learning models were developed and compared to predict stone-free status after PNL using clinical, radiological, and surgical variables derived from a large patient cohort. The findings demonstrate that the XGBoost-based model, in particular, achieved strong predictive performance, with high accuracy and ROC–AUC values in stone-free outcome prediction. These results indicate that, compared with the limited predictive capacity of conventional scoring systems, machine learning approaches offer superior potential for outcome prediction.

A key strength of this study is that it goes beyond the development of a high-performing model by rendering the decision-making process transparent through SHAP analysis. The quantitative demonstration of the effects of variables such as anatomical anomalies, sheath diameter, stone number, and stone location on stone-free outcomes allows model predictions to be interpreted in a manner consistent with clinical reasoning. This interpretability suggests that the proposed model is not only statistically robust but also clinically meaningful and potentially applicable as a decision-support tool.

In conclusion, this study shows that machine learning-based models for predicting stone-free status after PNL—particularly when supported by explainable artificial intelligence approaches—represent powerful tools that can contribute to individualized patient selection and surgical strategy planning. The proposed model holds promise for future integration into clinical decision-support systems and constitutes an important step toward improving success rates in PNL surgery.

## Figures and Tables

**Table 1:** Patient Characteristics and Overall Stone-Free Rate.

Variable	Value
Number of patients	2144
Age (years), mean ± SD (range)	49.3 ± 15.2 (18–90)
Male sex	61.0%
ASA I	28.7%
ASA II	16.8%
ASA III	54.5%
Complete stone-free rate	84.8%
Clinically insignificant residual fragments	8.0%
Clinically significant residual stones	7.1%

**Table 2:** Stone and Surgical Characteristics.

Variable	Value
Stone count, mean ± SD	2.16 ± 1.48
Stone surface area (mm^2^), mean ± SD	637.2 ± 432.8
Stone density (HU), mean ± SD	836.2 ± 151.0
Guy’s Stone Score I	35.5%
Guy’s Stone Score II	45.7%
Guy’s Stone Score III	7.4%
Guy’s Stone Score IV	11.4%
Partial/complete staghorn calculi	18.8%
Access sheath size (Fr), mean ± SD	24.4 ± 2.7
Operative time (min), mean ± SD	60.4 ± 23.9
Lower calyx access	70.4%
Second access tract required	4.8%
Anatomical anomalies	18.5%
Length of hospital stay (days), mean ± SD	1.99 ± 1.06

**Table 3:** Performance Metrics of the Models.

Model	Accuracy (95% CI)	Precision (95% CI)	Recall (95% CI)	F1-Score (95% CI)	ROC-AUC (95% CI)	CV Mean ± SD
**XGBoost**	0.9164 [0.8879, 0.9382]	0.9164 [0.8879, 0.9382]	0.9164 [0.8879, 0.9382]	0.9164 [0.8879, 0.9382]	0.9746 [0.9545, 0.9947]	0.9007 ± 0.0079
**Random Forest**	0.8901 [0.8586, 0.9153]	0.8904 [0.8590, 0.9155]	0.8901 [0.8586, 0.9153]	0.8901 [0.8586, 0.9153]	0.9634 [0.9394, 0.9874]	0.8797 ± 0.0092
**LightGBM**	0.9089 [0.8795, 0.9317]	0.9090 [0.8796, 0.9318]	0.9089 [0.8795, 0.9317]	0.9089 [0.8795, 0.9317]	0.9719 [0.9508, 0.9930]	0.9032 ± 0.0067
**AdaBoost**	0.8589 [0.8245, 0.8874]	0.8644 [0.8305, 0.8924]	0.8589 [0.8245, 0.8874]	0.8584 [0.8240, 0.8870]	0.9261 [0.8927, 0.9595]	0.8441 ± 0.0122

CI: Confidence interval; SD: Standard deviation.

## Data Availability

The datasets used and/or analyzed during the current study are available from the corresponding author on reasonable request.
